# Hoffa's fat pad tumours like: results of the arthroscopic resection

**DOI:** 10.11604/pamj.2015.20.187.4078

**Published:** 2015-02-27

**Authors:** Atif Mechchat, Hatim Abid, Hammou Nassreddine, Soufiane Bensaad, Elidrissi Mohammed, Shimi Mohammed, Elibrahimi Abdelhalim, Elmrini Abdelmajid

**Affiliations:** 1 Department of Orthopaedics and Trauma Surgery B4, UH Hassan II, Fez, Morocco

**Keywords:** Knee, Hoffa fat pad, tumours like, excision, arthroscopy

## Abstract

We performed a retrospective cohort study to increase awareness in orthopaedic community of this rare but interesting disease which is often misdiagnosed as meniscal pathology and to analyze present results of arthroscopic resection in seven patients. we retrospectively reviewed records from 2008-2012 and identified 7 patients with symptomatic Hoffa's fat pad impingement. The diagnosis was made by clinical exam, MRI imaging and verified arthroscopically. Of the 7 patients 2 were excluded due to receiving open resection. The remaining five underwent arthroscopic resection. Lysholm and American knee society scores were obtained pre and post operative and at final follow up. There was a significant improvement in their symptoms and function after the surgery at an average follow-up of 14 months. The one poor result was because of paresthesia over the distribution of the infrapatellar branch of the saphenous nerve after open resection. tumours like of the fat pad should be treated by arthroscopic resection because of less residual pain and less complication was found using arthroscopy. In case of high volume of tumours only open excision can provide complete excision.

## Introduction

Many tumours or tumours like can affect the Hoffa's fat pad. They usually occur in young active patients participating in activities involving repetitive microtrauma to the knee joint. Hoffa's disease was first described by Albert Hoffa in 1904 [1–3]. It's characterized by an impingement between the patellofemoral or femorotibial joints due to large volume tumours in the infrapatellar fat pad, causing chronic anterior knee pain [2]. The diagnosis was established by clinical signs, MR imaging and histological findings. The objective of this study is to increase awareness in orthopaedic community of this rare but interesting disease which is often misdiagnosed as meniscal pathology and to analyze present results of arthroscopic resection in seven patients. Hypothesis: arthroscopic resection of symptomatic Hoffa's fat pad tumours results in significant clinical improvement at final follow up.

## Methods

### Details

**Figure 1:**
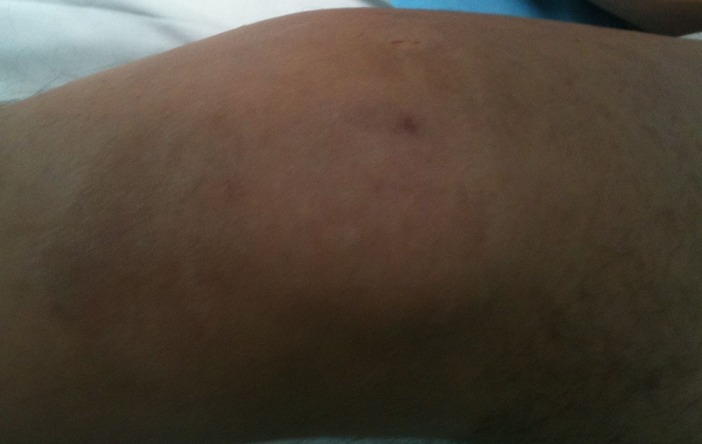
Chronic knee pain, mild anterior swelling and mild loss of knee extension

**Figure 2:**
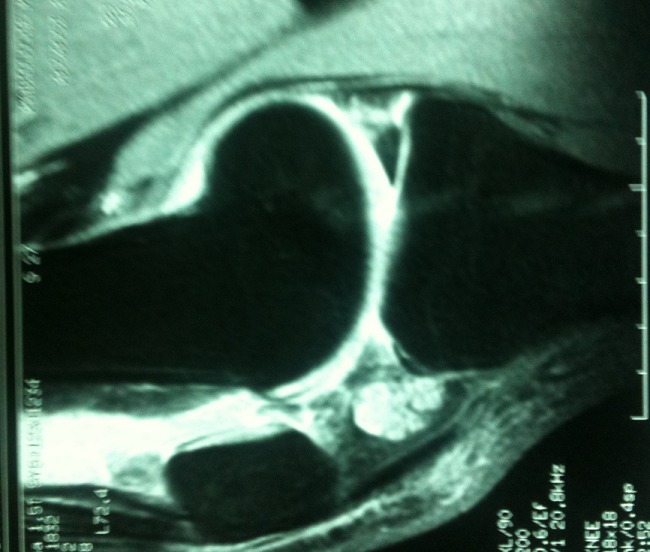
Abnormally increased volume of Hoffa's fat pad

We performed a retrospective analysis of a consecutive series of seven patients with the diagnosis of isolated Hoffa's tumour treated with arthroscopic resection of the infrapatellar fat pad between January 2008 and December 2012. The gender and age of the patients at presentation, the clinical features (Figure 1), investigations, preoperative diagnosis, operative findings, the anatomical site of origin of the tumour, the treatment and complications, including recurrence, were recorded. The Lysholm knee and American knee society were used for assessment preoperatively and at 3 months and at 1 year postoperatively, as well as at the conclusion of this study [4, 5]. The clinical features of the syndrome are well described and consist of pain and tenderness around the patellar tendon. Hoffa′s sign consists of applying pressure to the medial side of the fat pad, with a knee flexed. On extension the patient will experience pain as the fat pad is pinched intraarticularly [6]. Imaging All our patients had radiographic examinations of the knee radiographic examinations (anterior-posterior, lateral, and skyline views) and MR imaging 3 T to identify or exclude other knee pathology (Figure 2).

### Surgical technique

**Figure 3:**
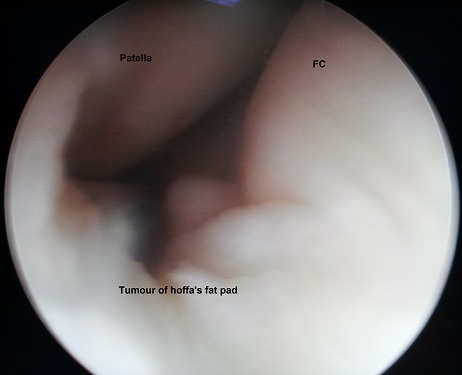
Arthroscopic view of the Hoffa's tumour like

**Figure 4:**
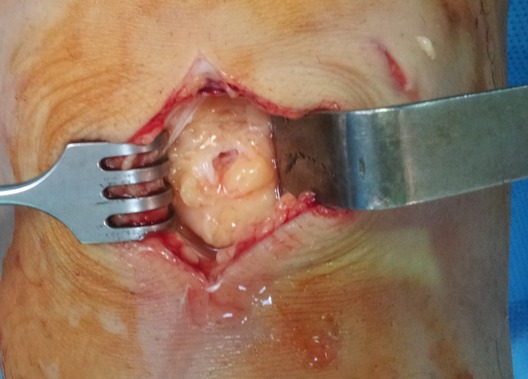
Open resection of Hoffa's fat pad

**Figure 5:**
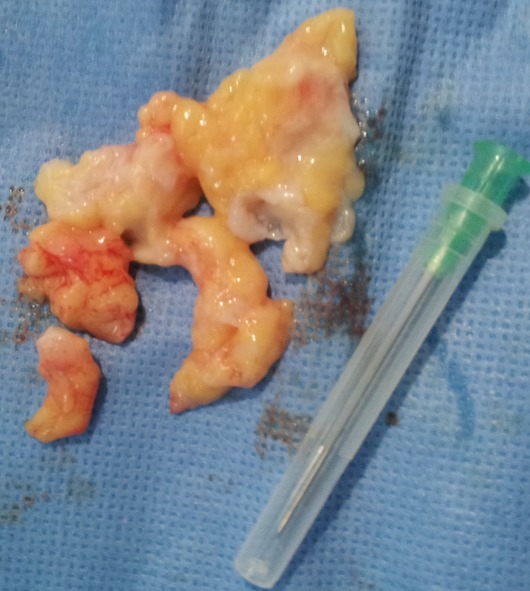
Excised Hoffa's fat pad in arthroscopy

The arthroscopy surgery was performed by using the standard anteromedial and anterolateral portals. Each knee compartment was inspected thoroughly and palpated using a blunt hook. The infrapatellar area is difficult to see in arthroscopic examination using the conventional anteromedial and anterolateral portals. We, therefore, made two accessory portals on the far medial and lateral sides of the conventional portals. Arthroscopic examination through these two accessory portals revealed a mass at the posterior border of the patellar tendon (Figure 3). There was requirement for a large arthrotomy (Figure 4) in two cases. The surrounding soft tissues were excised along with the tumors (Figure 5). Postoperatively the patients were placed on a vigorous physiotherapy program to strengthen the quadriceps power and regain range of movement. They were allowed full weight-bearing on the knee. Anti-inflammatory drugs were prescribed for the first 10 days postoperatively. The patients were reviewed using the Lyshlom knee and American knee society scores. This was compared with the preoperative score.

## Results

### Clinical features

In this study seven patients were diagnosed and treated for isolated tumours of Hoffa's fat pad. There were 5 females and 2 males. The average age at operation was 35 years with a range of 23- 44 (Table 1). The average duration of symptoms was 16 months with a range of 7 to 36 months. Five cases occurred in the right knee and two were in the left knee. Six patients had sustained minor injuries in the past but were unsure whether that precipitated their current symptoms, and just one denied any history of injury whatsoever.

**Table 1:** Clinical details of our series

Case number	Age (years)	Sex	Hoffa's test	Imaging modality	Histology	Treatment
1	44	F	+	MRI	Lipoma artborescens	Arthroscopic
2	31	M	+	MRI	Lipoma artborescens	Arthroscopic
3	45	M	-	MRI	Ganglion cyst	OE
4	53	F	+	MRI	Lesions of Hoffa's disease	Arthroscopic
5	38	F	-	MRI	Ganglion cyst	OE
6	26	F	+	MRI	Lesions of Hoffa's disease	Arthroscopic
7	18	F	-	MRI	Synovial cyst	Arthroscopic

### Radiological and pathological findings

Laboratory tests and radiographs were normal in all with no patella alta or patellar subluxation. MR imaging showed a mass-like lesion, encompassing the entire infrapatellar fat pad except for the anterior region just beneath the patellar tendon. The mass demonstrated a generalized heterogeneous hypointensity on T1- and T2-weighted images, with a poorly defined margin and amorphous internal clefts. Microscopy of the infrapatellar fat pad showed adipocyte necrosis, with the adipocytes surrounded by inflammatory cells showing mucoid degeneration and dystrophic calcification, with no mitotic activity. These findings supported the diagnosis of Hoffa's disease and were consistent with the MRI findings.

### Outcomes after excision

The average time to follow-up was 14 months [7]. The mean preoperative Lysholm knee score was 37.44, and it improved postoperatively to 82.20, 93.02, and 90.76 at 3 months, at 1 year, and at the latest follow-up, respectively. The average score for symptoms preoperatively was 32 and postoperatively was 46 (p < 0.001) (Table 2). The mean paired difference was 14 with a standard deviation of 6.3 and a 95% confidence interval of 9 to 18. The pre-operative score for function had a mean of 31 compared to a post-operative score of 46. The mean paired difference was 13 with a standard deviation of 7 and 95% confidence intervals of 9-18. This was significant (p < 0.001. Overall, six patients had an excellent result, and one had poor results because of paresthesia over the distribution of the infrapatellar branch of the saphenous nerve after open resection. American knee Society scores improved from 76 (range 17-100) pre-operatively to 96 (range 46-100) post-operatively with an improvement in functional scores from 92 (range 60-100) to 100.

**Table 2:** Clinical outcomes

	Mean preoperative score	Mean postoperative score	P value
**Lyshlom knee score**	37,44	90,76	<0.001
Score symptoms	32	46	--
Score function	31	46	--
**American knee society**	76	96	<0.001

## Discussion

The classically pain is present on the anteromedial aspect of knee joint like in Hoffa′s disease. [8] Knee joint has many fat pads which are extrasynovial but intracapsular. First one of the anterior fat pads is anterior suprapatellar (quadriceps), the second one is posterior suprapatellar (prefemoral), and the third one is infrapatellar (Hoffa, IFP) [1, 3, 9]. As per Ushiyama et al., HFP modulates chondrocyte function through production of cytokines like tumor necrosis factor (TNF), interleukin (IL)-1, basic fibroblast growth factor (bFGF), and vascular endothelial growth factor (VEGF) in the joint fluid [10]. Hoffa′s disease occurs in normal knees and Hoffa's syndrome is associated with hypertrophy of fat pad due to meniscal, capsular, or ligamentous lesions [1, 11]. Hoffas fat pad tumours like can be defined by their origin intrinsec or extrinsec to Hoffas fat pad. The intrinsec are characterized by pain, swelling, bruising, and flexion deformity of knee. The extrinsec are characterized by knee discomfort, recurrent hydroarthrosis, and joint weakening [1, 11, 12]. But in Hoffa's disease the Movements are usually less affected. Hoffa′s test is specific but difficult to elicit. Examiner presses on either side of the patellar tendon in flexed knee and the patient gradually extends the knee. Positive sign is pain, apprehension, or antalgic block during terminal extension [1, 12]. Clinical diagnosis poses difficulty as there are no definitive clinical symptoms and signs, but a palpable mass may suggest the diagnosis.

The main differential diagnosis involves meniscal lesions Metheny and Mayor [6] described the anatomy of the infrapatellar fat pad based on their cadaveric study and were the first investigators to report its arthroscopic resection, in 4 patients. Jacobson et al. [3] and Saddik et al. [13] described the MRI appearance of the infrapatellar fat pad in various disorders, stressing the importance of this diagnostic tool. A large fat pad or signals suggestive of inflammation seen on MRI are nonspecific and do not necessarily mean impingement. Our patients have been investigated by MRI to locate additional pathology, meniscale or ligamentary. The majority of solitary Hoffas fat pad tumours are benign. PVNS and intraarticular ganglia are the most commun in the literature [14], but in our series lipoma arborescens and synovial cyst were the two most commun pathologies In our series the diagnosis of fat pad impingement was made preoperatively and confirmed with arthroscopic and histological findings. From a technical point of view the fat pad was successfully resected arthroscopically in five cases. But complete excision was too difficult so we proceed by open excision in two cases for a ganglia cyst. Similar results are reported by Magi et al. but without detailed follow-up [11]. Overall there were one unsatisfactory result. In this particular case, and after an open excision, the patient had persistent symptoms behind the patella tendon that caused a mild limitation of their functions in addition to paresthesia of the infrapatellar branch.

## Conclusion

Begnin tumours should be achieved by arthroscopy rather than by open resection because it results in significant clinical improvement at final follow up. In case of high volume of tumours only open excision can provide complete excision.
